# Temporal dynamics of the lung and plasma viromes in lung transplant recipients

**DOI:** 10.1371/journal.pone.0200428

**Published:** 2018-07-06

**Authors:** Maia Segura-Wang, Irene Görzer, Peter Jaksch, Elisabeth Puchhammer-Stöckl

**Affiliations:** 1 Center for Virology, Medical University of Vienna, Vienna, Austria; 2 Division of Thoracic Surgery, Medical University of Vienna, Vienna, Austria; Institut National de la Recherche Agronomique, FRANCE

## Abstract

The human virome plays an important role for the clinical outcome of lung transplant recipients (LTRs). While pathogenic viruses may cause severe infections, non-pathogenic viruses may serve as potential markers for the level of immunosuppression. However, neither the complexity of the virome in different compartments nor the dynamics of the virus populations posttransplantation are yet understood. Therefore, in this study the virome was analyzed by metagenomic sequencing in simultaneously withdrawn bronchoalveolar lavage (BAL) and plasma samples of 15 LTRs. In seven patients, also follow-up samples were investigated for abundance and dynamics of virus populations posttransplantation. Five eukaryotic and two prokaryotic virus families were identified in BAL, and nine eukaryotic and two prokaryotic families in plasma. Anelloviruses were the most abundant in both compartments, followed by Herpes- and Coronaviruses. Virus abundance was significantly higher in LTRs than in healthy controls (Kruskal-Wallis test, *p*<0.001). Up to 48 different anellovirus strains were identified within a single LTR. Analyses in the follow-up patients revealed for the first time a highly complex and unique dynamics of individual anellovirus strains in the posttransplantation period. The abundance of anelloviruses in plasma was inversely correlated with that of other eukaryotic viruses (Pearson correlation coefficient *r* = −0.605; *p<*0.05). A broad spectrum of virus strains co-exists in BAL and plasma of LTRs. Especially for the anelloviruses, a high degree of co-infections and a highly individual and complex dynamics after transplantation was observed. The biological impact of these findings and their relation to clinical variables remain to be elucidated by future analyses.

## Introduction

The human microbiome, *i*.*e*. the complete community of microorganisms (including eukaryotes, archaea, bacteria and viruses) living in the human body, has an important influence on human health and disease [1,2]. So far there is only limited insight in the viral part of the microbiome, the human virome. Viruses may cause severe infections in immunocompromised individuals, such as transplant recipients [3–6]. Lung transplant recipients (LTRs) receive high level immunosuppressive therapy. Therefore, severe infections with viruses of the *Herpesviridae* family as Cytomegalovirus (HCMV), or with respiratory viruses may occur and are a major cause of morbidity and mortality after lung transplantation [3,7]. Also non-pathogenic viruses replicate at high levels in immunosuppressed persons. Especially Torque Teno Viruses (TTV) and other members of the *Anelloviridae* family, which are highly prevalent in the human population, extensively replicate under immunosuppression [5,8]. These non-pathogenic viruses have recently gained particular interest as first data shows that the TTV-level may reflect the extent of immunosuppression after lung transplantation and may be a useful marker for optimizing immunosuppressive therapy [9–12].

Additionally, there is first evidence that the human virome may undergo substantial temporal changes [13,14] and that it may differ considerably between different human body compartments [2]. However, the detailed dynamics of viral populations after transplantation has so far not been clearly assessed.

The aim of the present study was therefore to analyze in detail the virome of lung and blood in LTRs by using a metagenomic approach and to assess the temporal dynamics of the virome in both compartments after lung transplantation.

## Materials and methods

### Study population

In this retrospective study, fifteen lung transplant recipients (LTRs) transplanted at the Medical University of Vienna between 2013 and 2015 were investigated. This study was approved by the Ethics Committee of the Medical University of Vienna under EK-number 1880/2016. All transplant recipients gave their written informed consent and all data were fully anonymized before the analyses were performed. None of the transplant donors were from a vulnerable population and all donors or next of kin provided written informed consent that was freely given.

For seven of these patients follow-up investigations were performed on 24 paired bronchoalveolar lavage (BAL) and plasma samples taken on the same day (2 to 6 samples/patient) within the first year post-transplantation. From eight other LTRs, paired BAL and plasma samples were taken at a single time point after transplantation. Patients received immunosuppressive treatment with induction with alemtuzumab (Campath-1H) and maintenance therapy with tacrolimus (Prograf) and corticoids (Prednisolone). Additionally, all patients received antibiotic prophylaxis for two weeks, anti-fungal prophylaxis with aerosolized amphotericin B for three months, and HCMV-prophylaxis consisted of four doses of intraveneous HCMV hyperimmunglobulin during the first month and Val/ganciclovir administered for at least 3 months post-transplantation. As controls, ten blood samples from seven healthy individuals were included. For three of them a second sample was taken 57, 162 and 106 days after the first sample.

### Viral enrichment

One ml BAL or 500ul plasma were centrifuged for 5min at 1200g. Volumes were adjusted to 1.5ml with PBS and filtered through 0.45μM filters (Sartorious). To remove cell-free DNA and RNA, the supernatant was treated with RNase A (Qiagen) and TURBO DNase (Ambion) to final concentrations of 0.77mg/ml and 20Units/ml, respectively, for 1h at 37°C, and deactivated by Protease (Qiagen) treatment for 30min at 37°C.

### Nucleic acid extraction

Total nucleic acids were extracted using 1000ul of the filtered and nuclease treated material, with the automated NucliSENS EasyMAG system (BioMérieux).

### Metagenomic sequencing

For each extracted sample, RNA and DNA were amplified by random primer amplification in separate reactions and pooled together before sequencing. RNA was first reverse transcribed using a random octamer linked to an anchor sequence ATCGTCGTCGTAGGCTGCTCNNNNNNNN [15–18]. In short, 5μl of extracted eluate and 5μM random primers were incubated for 5min at 65°C. Then 20μl total volume reactions were set up as described in Lewandowska *et al*. [18]. The resulting cDNA was denatured for 2min at 94°C, cooled 5min at 10°C and used for second-strand synthesis. Similarly, the DNA from the extracted eluate was randomly amplified by using 5μl of template. The resulting DNA derived from both RNA and DNA templates was subsequently amplified in separate reactions by PCR using 4μl of input DNA from the previous step, 0.25μM dNTP mix (Promega), 2mM MgCl_2_, 1x AmpliTaq Gold Buffer II (Applied Biosystems), 0.05U/μl AmpliTaq Gold Polymerase (Applied Biosystems) and 1μM of an anchor specific primer for a total volume of 50μl [18]. The products were quantified by Qubit dsDNA BR Assay (Invitrogen) and diluted to 0.4ng/μl. Equal amounts of the amplified and diluted cDNA and DNA were pooled together at 0.4ng/μl, and used as input for library preparation using the NexteraXT Library Preparation kit (Illumina). Samples were sequenced on an Illumina MiSeq platform, single-read 150bp. BAL and plasma samples from the seven follow-up LTRs were processed in duplicate. For the rest of the samples, single experiments were performed.

### Data analysis

Raw reads were quality trimmed using Seqtk (https://github.com/lh3/seqtk). To remove contaminant host sequences, reads were sequentially aligned to the human reference genome (GRCh38), and to sets of bacterial and fungi genomes using BWA-MEM [19]. We then mapped the decontaminated reads with Bowtie2 v.2.2.9 [20] to 249 verified complete anellovirus genome sequences from NCBI, including strains of the 5 known main TTV genogroups and TTV-mini and -midi strains. The rest of the reads were mapped against 49087 viral complete genomes obtained from the NCBI GenBank [21] nuccore database [15,18]. We estimated the abundance of each viral strain in every individual, expressed as reads per million. Given the high correlation between duplicates (Pearson correlation coefficient *r* = 0.898; *p<*0.001), we decided to use the average abundance of each strain for further analyses. Reads not mapping to any of the above genomes were considered to be of unknown origin. The Shannon diversity index (H) of anellovirus strains was estimated for each sample based on the abundance of each strain and using the Vegan R package [22].

### Phylogenetic analysis

Phylogenetic trees were built based on the reference anellovirus sequences, according to the strains identified in BAL and plasma, by doing a multiple sequence alignment with ClustalW implemented in MEGA7 [23], followed by construction of a neighbor-joining tree using default parameters. The iTOL software was use for visualizing and annotating the phylogenies [24].

### Statistical analyses

Statistical analyses and plots were made using the software environment R, version 3.2.2 [25].

## Results

### Virome content in the BAL and plasma of lung transplant recipients

First we aimed to characterize the viral content in simultaneously withdrawn BAL and plasma samples in LTRs. Therefore, virome analysis using metagenomics sequencing was performed in all 32 paired BAL and plasma samples withdrawn from the 15 LTRs included in the study. More details about the patients and samples are shown in Table 1. The average raw total number of reads per sample was 703242 (±395169). After quality control and filtering, an average of 39.6% (±26.6) reads remained for further analysis. Of these reads, 37.2% (±25.9) aligned to the human genome, 10.8% (±7) reads aligned to bacteria and 0.36% (±0.05) to fungi and these were all removed prior to further analyses.

**Table 1:** General information of the 15 lung transplant recipients included in this study.

Patient	Age (years)	Underlying Disease^1^	Gender	Time Point	DaysPostTx^2^
F1	59	COPD	M	T1	38
				T2	65
				T3	163
				T4	219
				T5	284
				T6	345
F2	21	CF	F	T1	58
				T2	85
				T3	172
				T4	378
F3	19	CF	F	T1	67
				T2	195
F4	19	CF	F	T1	67
				T2	193
F5	28	CF	F	T1	96
				T2	131
				T3	225
F6	50	COPD	F	T1	36
				T2	67
				T3	96
				T4	173
F7	59	COPD	F	T1	79
				T2	93
				T3	171
P1	51	COPD	F	T1	94
P2	57	COPD	F	T1	99
P3	22	CF	F	T1	92
P4	63	COPD	M	T1	79
P5	37	CF	F	T1	82
P6	49	COPD	M	T1	92
P7	23	CF	M	T1	75
P8	50	IPF	M	T1	88

^1^CF: Cystic fibrosis, COPD: Chronic obstructive pulmonary disease, IPF: Idiopathic pulmonary fibrosis.

^2^Days post-transplantation.

**Fig 1:**
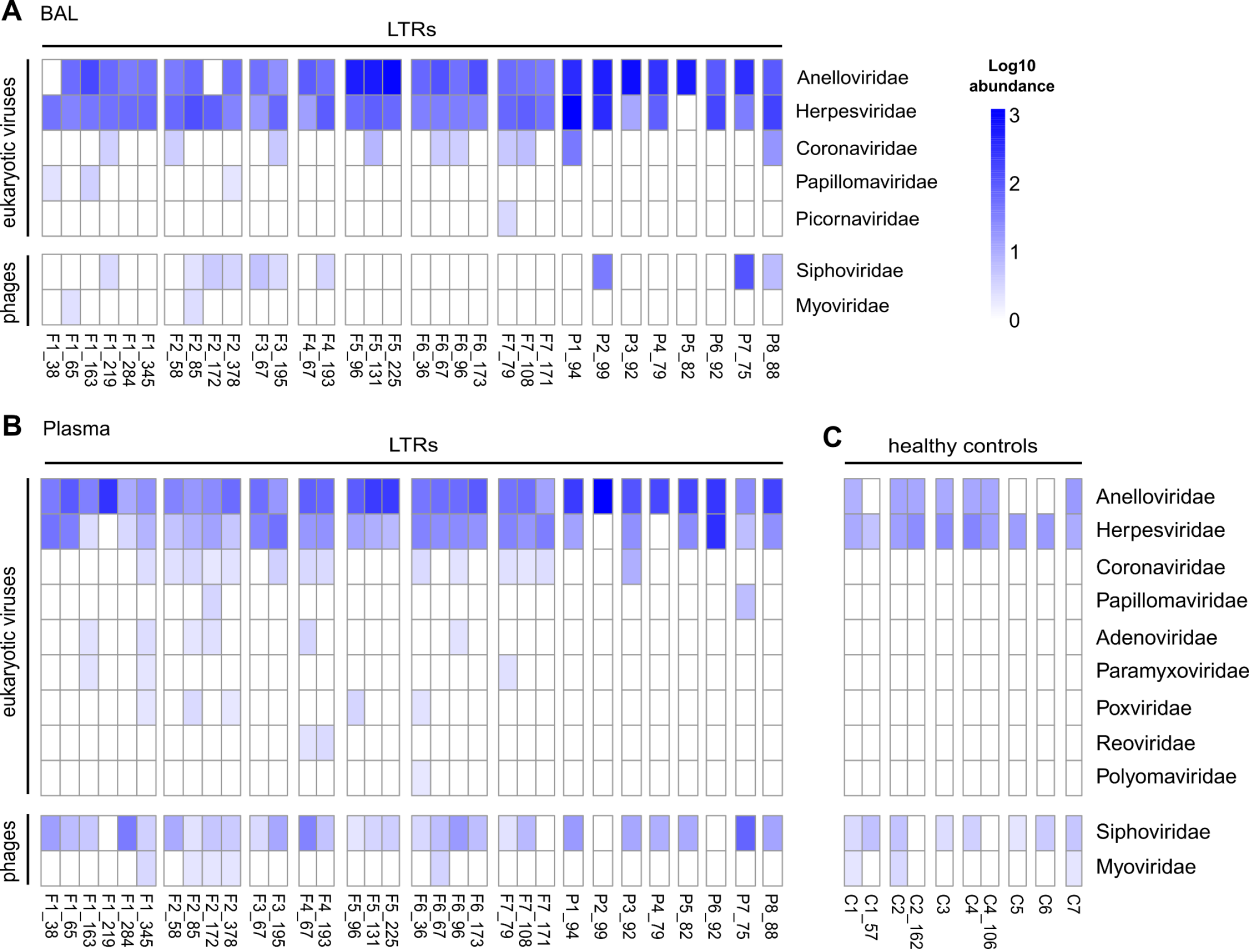
Viral families identified in 15 lung transplant recipients. (A) Viral families identified in BAL samples, and (B) plasma samples obtained simultaneously at different time points after transplantation (F1-F7: LTRs with follow up samples; P1-P8: LTRs tested at only one time point). (C) Viral families identified in 7 healthy controls (C1-C7). Numbers after the patient identifier indicate days after transplantation for LTRs; and days between first and second sample for the healthy controls. Log10 abundance in reads per million.

All cleaned reads (19.9% ±18.8 of the total sequencing reads) were aligned to a database of complete viral genomes. On average, 3.11% (±2.9) of reads were aligned to a virus strain. We identified seven different viral families in the BAL and 11 in the plasma samples (Fig 1A and 1B and S1 Table). Most viruses identified showed low abundances of less than 10 reads per million, and were detected only in few patients. Eukaryotic viruses identified in both, BAL and plasma, belonged mainly to the *Anelloviridae*, *Herpesviridae* and *Coronaviridae* families. In both body compartments of LTRs prokaryotic viruses of the *Siphoviridae* family, including *Propionibacterium* phages and *Pseudomonas* phages, and of the *Myoviridae* family, including *Staphylococcus* phages and *Streptococcus* phages, were identified. In plasma of healthy controls, only two eukaryotic viral families were identified, *Anelloviridae* and *Herpesviridae* (Fig 1C) as well as the two mentioned prokaryotic families. In the patients with follow-up samples, we observed changes in virus populations over time (Fig 1A and 1B).

### Anellovirus abundance in BAL and in plasma

**Fig 2:**
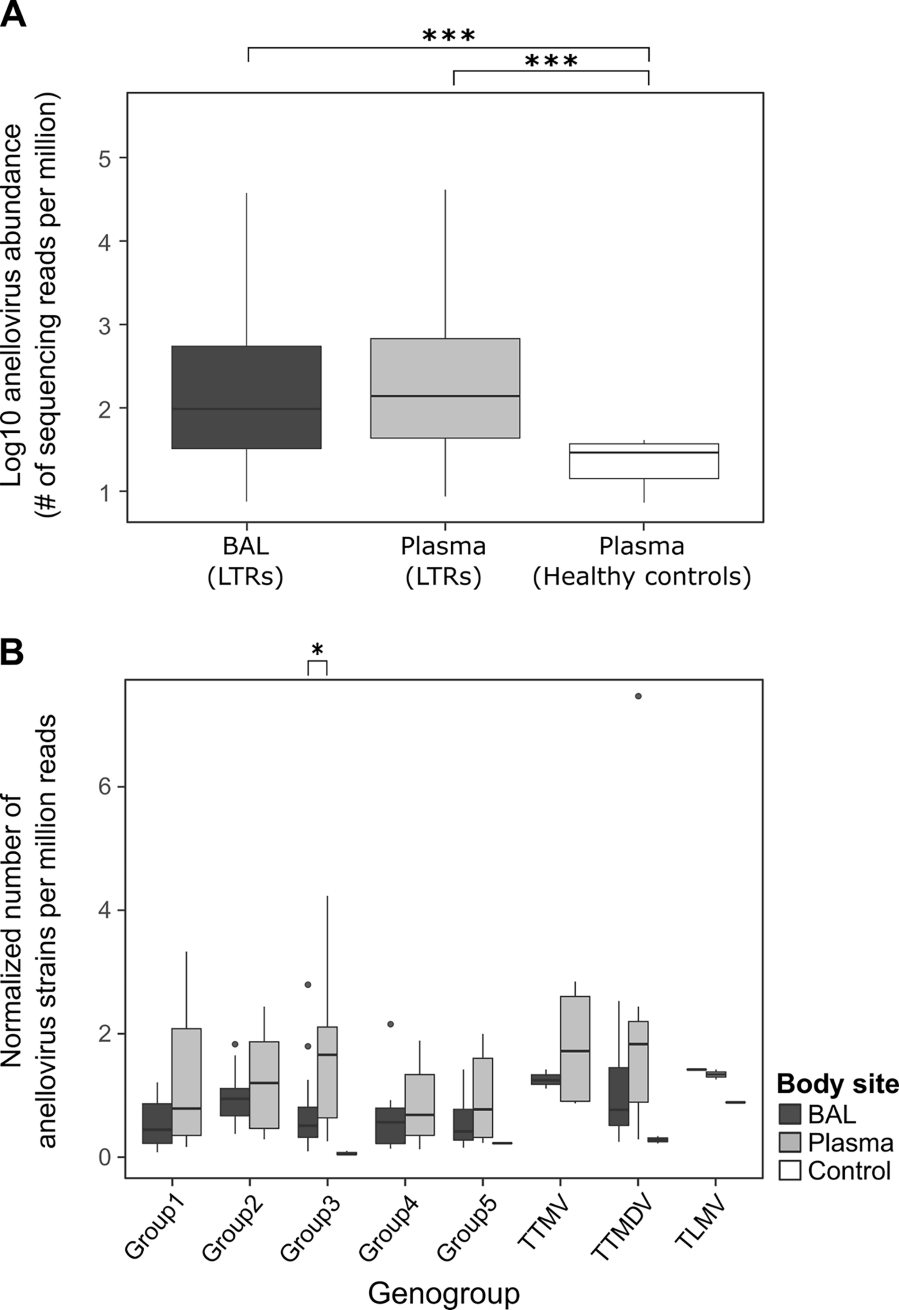
Abundance of anelloviruses in different body compartments and number of strains by genogroup. (A) Abundance of anelloviruses (number of sequencing reads per million) in BAL and plasma of 15 LTRs and in plasma of healthy controls (***:*p*<0.001; Kruskal-Wallis with post-hoc test). (B) Number of anellovirus strains in each genogroup normalized by the total number of reads sequenced for each sample and the total number of different strains identified per genogroup (*:*p*<0.05; Wilcoxon rank sum test).

As recent investigations showed that the plasma virus load of distinct anelloviruses may be useful as a potential marker for the level of immunosuppression in LTRs [9], we further focused on this virus group and analyzed in more detail the anellovirus content in all samples of LTRs and healthy controls. The overall abundance of anelloviruses was similarly high in BAL and blood of the 15 patients, and significantly higher in both BAL and blood of LTRs compared to plasma of the healthy controls (*p*<0.001 and *p*<0.001 respectively; Kruskal-Wallis with post-hoc test) (Fig 2A). Similar numbers of different strains per genogroup were found in the patient BAL and plasma samples. Only for genogroup 3 a significantly higher number of strains was found in plasma than in BAL (*p*<0.05; Wilcoxon rank sum test) (Fig 2B). No significant correlation between the total number of reads/sample and the number of anellovirus strains identified was observed. This confirms that the identification of strains was not dependent on the sequencing depth (S1 Fig).

### Diversity and dynamics of anelloviruses in the follow-up after lung transplantation

**Fig 3:**
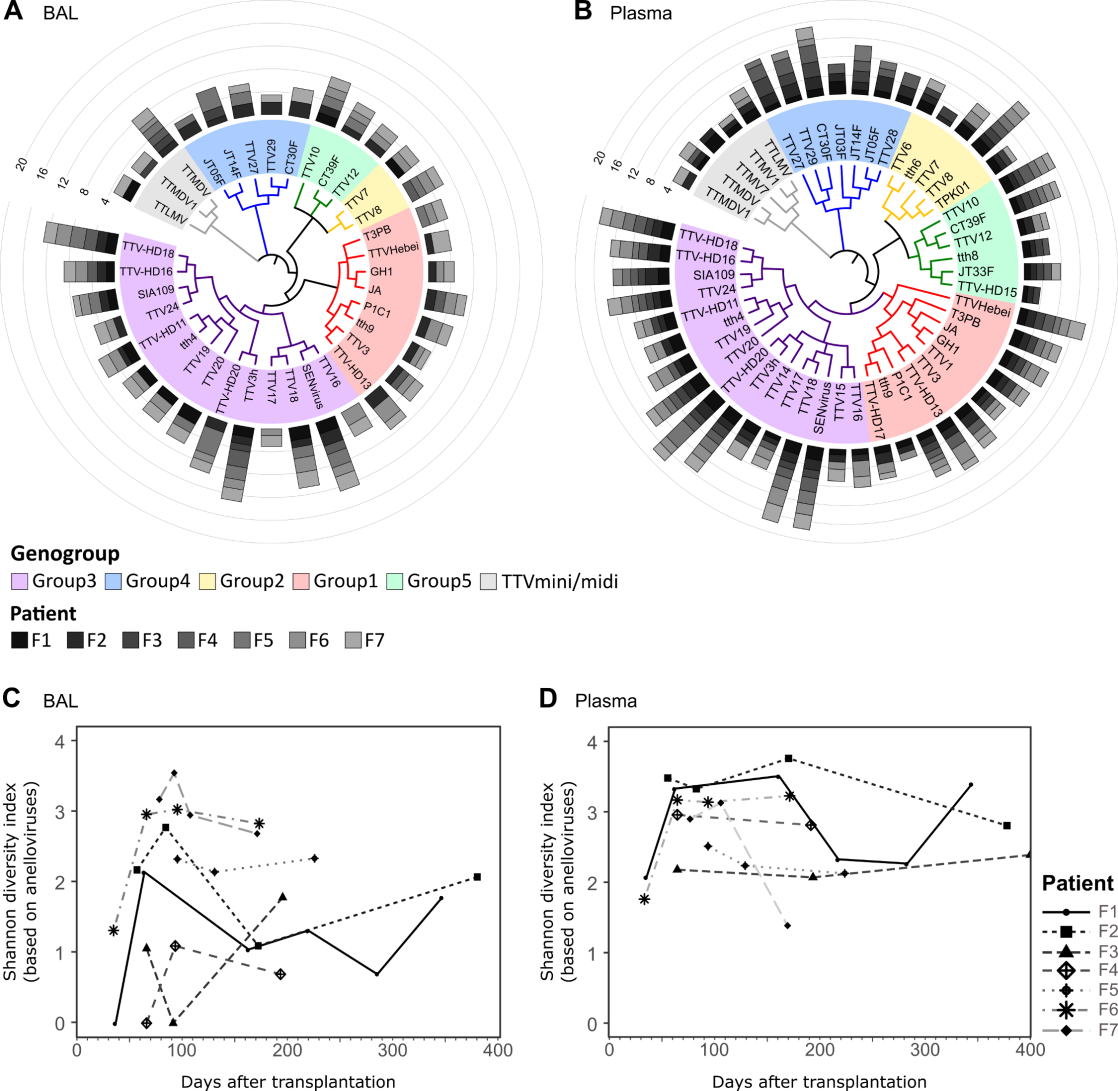
Abundance and diversity of anellovirus strains in BAL and plasma. (A) and (B) Average abundance of anellovirus strains found over all time points in BAL and plasma samples of LTRs. The strains are shown according to phylogenetic similarity and grouped by genogroup. Bar height indicates Log10 abundance (reads per million). (C) Shannon diversity indexes of LTR BAL samples based on TTV strains. (D) Shannon diversity indexes of LTR plasma samples based on TTV strains.

We further assessed in detail how the distribution of the different anellovirus strains changes over time in the seven LTRs, with available follow-up (Table 1). The distribution of anellovirus strains identified at the different time points after transplantation in each LTRs is presented in Fig 3A and 3B. The data show that each LTR carries an individual and highly diverse set of anellovirus strains in both body compartments. The strains with the highest abundance over all BAL samples were SEN virus and TTV-3h, both of genogroup 3, and in all plasma samples, TTV18 and SEN virus, also both genogroup 3. As shown in Fig 3C and 3D the diversity (measured by the Shannon diversity index) was highly variable between the patients and time points, and was significantly higher in plasma than in BAL (*p*<0.001; Wilcoxon rank sum test. S2 Fig).

**Fig 4:**
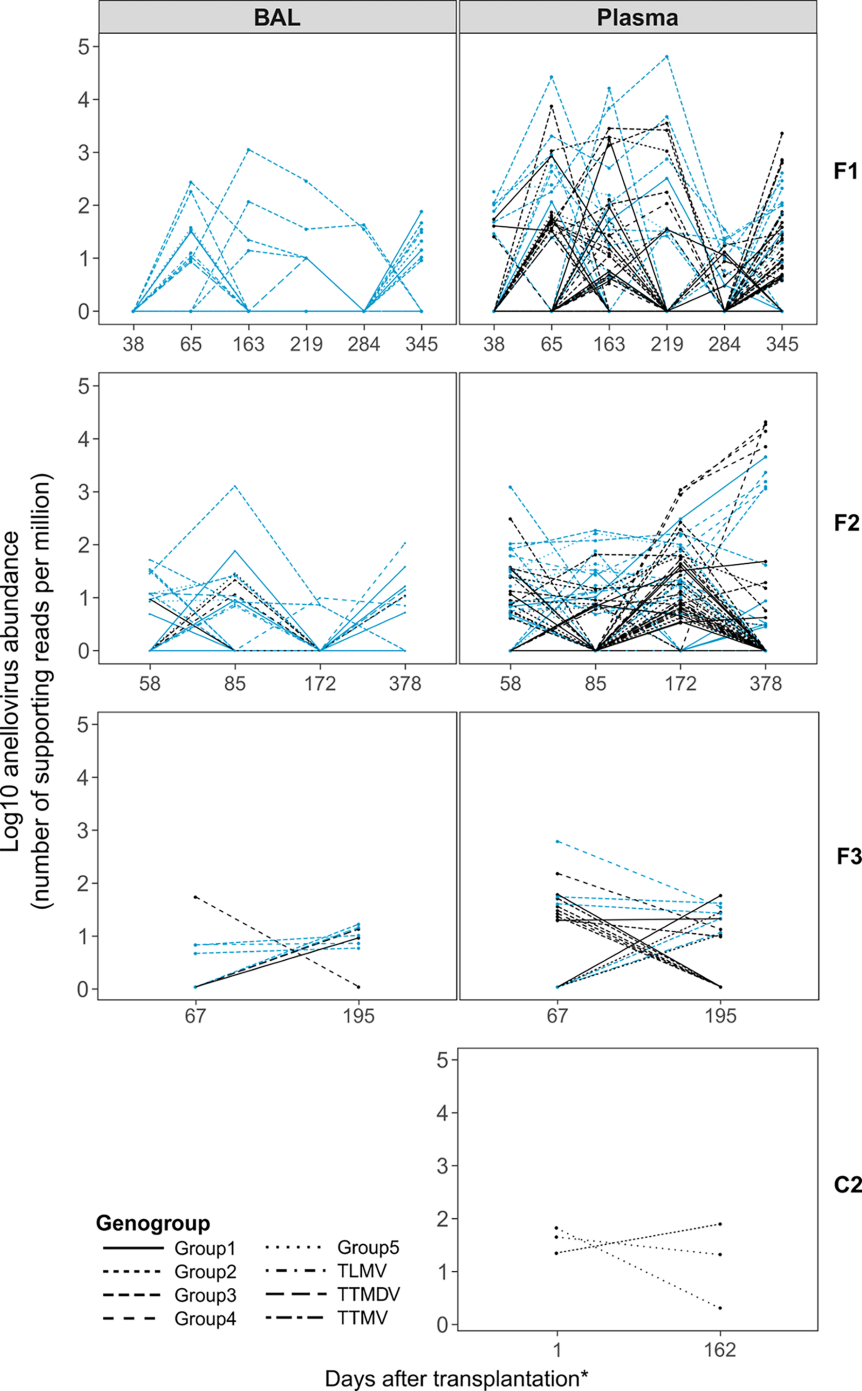
Within patient anellovirus isolates present at each time point after transplantation in BALs and plasma samples of LTRs (F1-F3). C2 is a healthy control sample. *For C2, y-axis indicates day at which sample was taken. Blue lines: anellovirus strains detected simultaneously in BAL and plasma. Black lines: anellovirus strains present in only one body compartment.

To study the within-patient dynamics of *Anelloviridae* strains over time we characterized the abundance of the individual anellovirus isolates present in both body compartments of the patients at different time points. The data for each patient is depicted in Fig 4 and S3 Fig. Each patient showed a wide variety and complex dynamics of *Anelloviridae* strains over time. Most anellovirus strains present in BAL were also detectable in the plasma samples withdrawn on the same day, while in the plasma samples mostly additional anellovirus strains were detected. In total, up to 28 different anellovirus isolates were identified in BAL and up to 48 in plasma samples. A detailed overview of the number of different isolates in BAL and plasma of each patient is shown in S4 and S5 Figs. Seven anellovirus strains were detected in all LTRs at least once: SEN virus, TTV18, TTV20, TTV16, TTV-HD20, TTV-HD18 (all genogroup 3) and Torque-Teno-midi virus. Of all strains observed, eight were detected exclusively in plasma and 53 in both compartments. No strains were exclusively detected in BAL.

We have further analyzed the anellovirus content in the plasma of three healthy control individuals at two different time points. As shown in Fig 4 and S6 Fig the abundance of anellovirus strains in these patients was low and showed limited dynamics.

### Relation between the abundance of anelloviruses and other eukaryotic viral families

**Fig 5:**
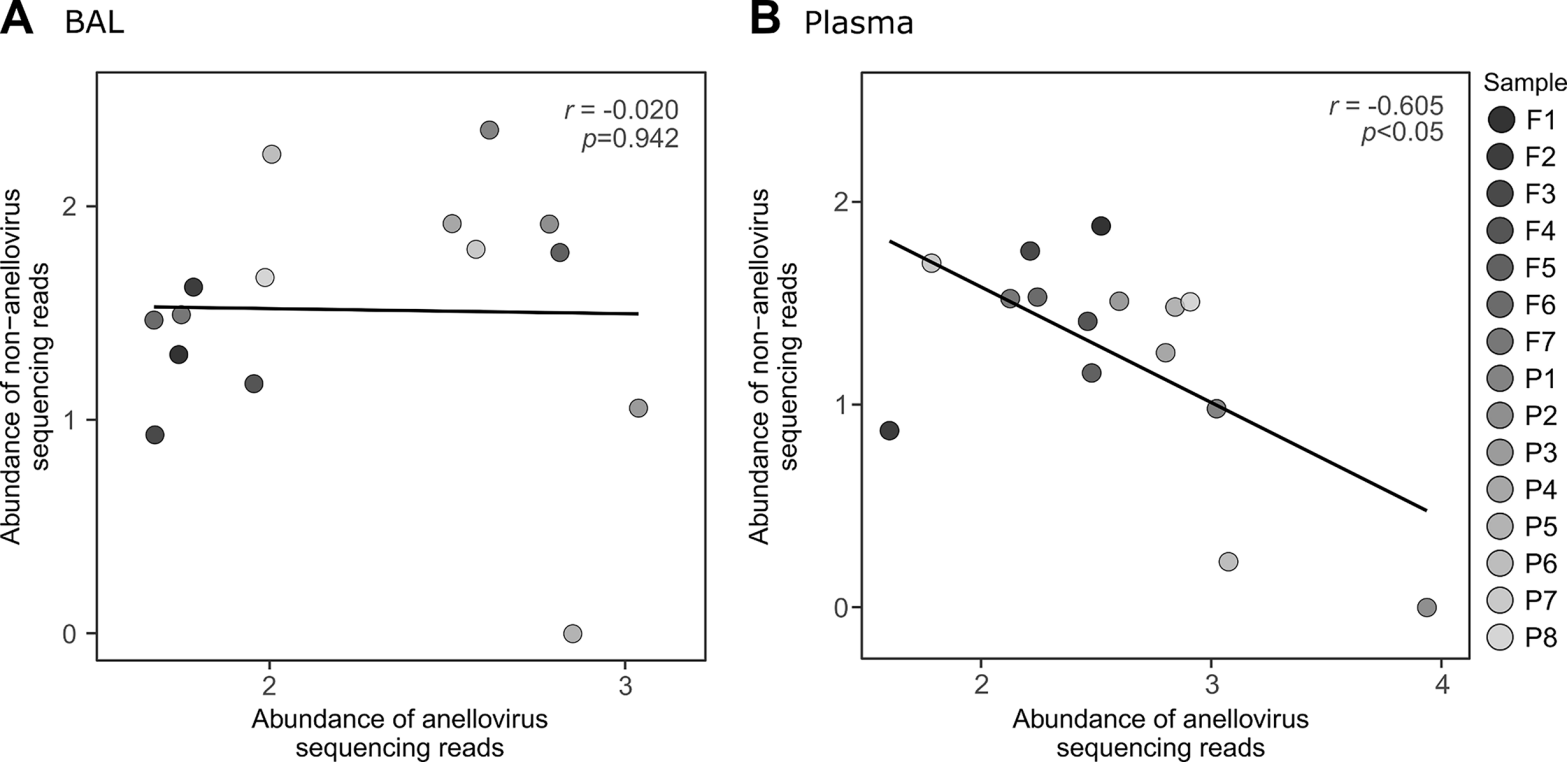
Relation between the abundance of anelloviruses and of other eukaryotic viruses. (A) and (B) Abundance of anelloviruses (Log10) versus the abundance of other eukaryotic viruses (non-anelloviruses) in BAL and plasma of 15 LTRs, respectively. Only one sample per patient was used for this analysis, for the time points closer to 87 days after transplantation.

To evaluate the relation between the abundance of anelloviruses and the presence of other eukaryotic viral families in the same compartment, we assessed the relative abundance of both viral groups in all 15 LTRs. To have comparable time points posttransplantation, only one sample per patient was used for this analysis, taken between day 65 and day 96 posttransplantation. No significant correlation between the abundance of anelloviruses and other eukaryotic viral families was found in BAL (Fig 5A), while a significant negative correlation was observed between the abundances of anellovirus and other eukaryotic viruses in plasma of LTRs (Pearson correlation coefficient *r* = −0.605; *p<*0.05) (Fig 5B).

### Anellovirus content, underlying disease and gender

We finally investigated if the virome content of the 15 LTRs is associated with the patients’ underlying disease, as shown in Table 1. No significant difference in anellovirus diversity was found in BAL or plasma between patients with COPD or CF (*p*>0.05; Wilcoxon rank sum test) (S7A Fig). Also, no significant difference in diversity was found between male and female LTRs (*p*>0.05; Wilcoxon rank sum test) (S7B Fig).

## Discussion

In this study we show that there is a highly complex virome in BAL and blood in patients after lung transplantation. In both compartments various eukaryotic viruses were detected, and similar to what was shown previously, always anelloviruses were the most abundant ones [26,27], followed by members of the *Herpesviridae* family [5,26]. This is of particular importance given that herpesviruses contribute significantly to clinical complications after transplantation, by causing severe infections and by serving as trigger for rejection processes [28]. Other respiratory viruses identified especially in the BAL, such as coronaviruses and rhinoviruses, are important pathogens which may also contribute to rejection of the transplanted lung [29,30]. In both body compartments also prokaryotic viruses, especially *Myoviridae* and *Siphoviridae* (Caudovirales order) were found. Similarly, DeVlaminck *et al*. [5] found that *Caudovirales* were highly abundant after transplantation. While different studies show that presence and variety of different bacteriophages in the gut may contribute to human health or disease [31] such clinical effects are not clear for bacteriophages found in blood or lung. In the plasma samples, sequencing reads aligning to some viral families were identified, whose clinical significance in these patients is not fully understood. Given the low number of reads, their presence should be confirmed in further studies.

Viruses of the *Anelloviridae* family are highly prevalent in the human population. Overall these viruses are considered as non-pathogenic [32]. However, anelloviruses, and in particular TTV, have gained substantial interest in the last years as it was shown that the plasma TTV-load may reflect the level of immunosuppression after transplantation. Also the present findings showed a clearly lower TTV abundance in healthy individuals compared to LTRs who receive drug-induced immunosuppression. The TTV-load, measured by a PCR assay covering the vast majority of TTV strains, is currently considered as a potential marker for guiding immunosuppressive drug therapy after transplantation [8,33,34]. However, so far no detailed and comprehensive analyses of the anellovirus populations present in the transplanted host and its dynamics over time have been performed. By using NGS and metagenomic analyses we here reveal for the first time that there is a highly complex pattern of anellovirus populations and kinetics in different body compartments.

A number of new aspects were now made visible by using these technologies. First, it was shown that the anellovirus populations were diverse between lung and blood compartment and that the strain population was always much more complex in blood than in the BAL. Further analyses are required to clarify whether individual virus strains are better adapted to a particular environment within the body, as has been reported for bacterial populations [35]. Similar to other studies, TTV strains of genogroup 3 were the most common in both compartments [36,37], which raises the question whether these anellovirus strains are better adapted to the human host than others or whether they circulate more frequently in most populations.

Second, our data show that overall there is an enormously high level of co-infections with different anellovirus strains in LTRs, which is not seen in the healthy controls. It is unclear from where these strains derive. They may be present at undetectable levels already before immunosuppression, or they are acquired before transplantation during the patients’ course of CF or COPD. Also a part of the strain populations may derive from anellovirus strains transferred to the host by the donor lung [27]. In any case, these data further suggest that there is little or no cross immunity between the different anellovirus strains [36].

Third, our results indicate for the first time that the anellovirus strain populations are not only very complex at each single time point, but they also show an extremely high degree of variation in the follow-up dynamics. So far it is unclear what drives these dynamics and leads to such large changes in the individual level of replication of the individual virus strains over time. Also for HCMV it was revealed in the last years that different genotypes show a complex and not understood replication pattern over time after lung transplantation [38,39]. It remains to be evaluated whether host factors or virus specific factors drive such dynamics and what this may mean for the clinical development of the patients. Interestingly, Blatter *et al*. [34] recently reported that TTV from different genera are associated with different outcomes in pediatric lung transplantation. It remains to be investigated whether particular strains are also associated with acute rejection or other longer-term outcomes.

Interestingly, the present virome analyses revealed that there is a significant negative correlation between the abundance of anelloviruses and other eukaryotic viral families in the plasma of LTRs. This is in agreement with the fact that we and others have found previously that the TTV-load, may decrease in patients when other viruses as HCMV (personal unpublished data) or hepatitis E virus replicate at high levels [11]. We speculate that the inflammatory response against other viruses eventually also may impair TTV replication [11,40]. However, further studies are needed to analyze this aspect more in detail, especially regarding the potential use of TTV as a marker for immunosuppression.

In conclusion, our data show that there is a complex virome present in lung and blood of LTRs, which consists of an enormous variety of virus strains and changes substantially in the post-transplant follow-up. Further analyses are needed to elucidate whether and to which degree the individually different and changing complexity of the virome may have an impact on the individual clinical development of patients after lung transplantation.

## Supporting information

S1 Table Total number of sequencing reads per viral strain identified in BAL and plasma samples of LTRs and healthy controls. (XLSX)

S1 Fig Correlation between the total number of anellovirus strains identified and the total number of reads sequenced per sample. (Pearson correlation coefficient *r* = 0.172; *p* = 0.137).
(PDF)

S2 Fig Shannon diversity index for BAL and plasma samples from 7 follow up LTRs. (**p<0.001; Wilcoxon rank sum test).
(PDF)

S3 Fig Anellovirus dynamics over time in 4 follow up lung transplant recipients (F4-F7). In blue the strains that occur both in BAL and plasma are displayed.
(PDF)

S4 Fig Number of anellovirus strains per patient and per time point identified in the BAL of LTRs. The number of strains in each intersecting set is shown as vertical barplots and the total number of strains identified per time point is shown in blue horizontal barplots.
(PDF)

S5 Fig Number of anellovirus strains per patient and per time point identified in the plasma of LTRs. The number of strains in each intersecting set is shown as vertical barplots and the total number of strains identified per time point is shown in blue horizontal barplots.
(PDF)

S6 Fig Anellovirus dynamics over time in two plasma samples obtained from healthy controls. (PDF)

S7 Fig Anellovirus diversity. Anellovirus diversity (measured by Shannon diversity index) in LTRs in relation to (A) underlying disease (CF: cystic fibrosis; COPD: chronic obstructive pulmonary disease), or (B) gender.
(PDF)
