# Supplementary material for: Temporal dynamics of the lung and plasma viromes in lung transplant recipients
Source: PLoS One. 2018 Jul 6;13(7):e0200428. doi: 10.1371/journal.pone.0200428 (PMC6034876; doi:10.1371/journal.pone.0200428)
Supplement: S1 Fig — (Pearson correlation coefficient r = 0.172; p = 0.137). (PDF) [file pone.0200428.s002.pdf]

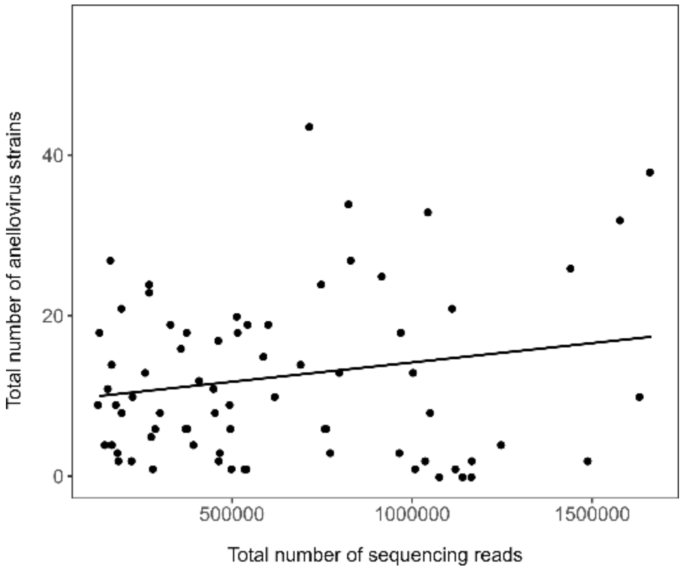

Figure S1. Correlation between the total number of anellovirus strains identified and the total number of reads sequenced per sample (Pearson correlation coefficient r=0.172; p=0.137).
