# Supplementary material for: Temporal dynamics of the lung and plasma viromes in lung transplant recipients
Source: PLoS One. 2018 Jul 6;13(7):e0200428. doi: 10.1371/journal.pone.0200428 (PMC6034876; doi:10.1371/journal.pone.0200428)
Supplement: S2 Fig — (**p<0.001; Wilcoxon rank sum test). (PDF) [file pone.0200428.s003.pdf]

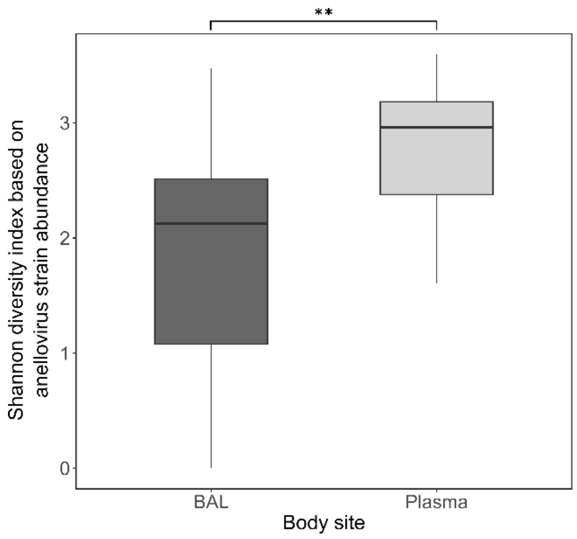

Figure S2. Shannon diversity index for BAL and plasma samples from 7 follow up LTRs (**p<0.001; Wilcoxon rank sum test).
