# Supplementary material for: Temporal dynamics of the lung and plasma viromes in lung transplant recipients
Source: PLoS One. 2018 Jul 6;13(7):e0200428. doi: 10.1371/journal.pone.0200428 (PMC6034876; doi:10.1371/journal.pone.0200428)
Supplement: S3 Fig — In blue the strains that occur both in BAL and plasma are displayed. (PDF) [file pone.0200428.s004.pdf]

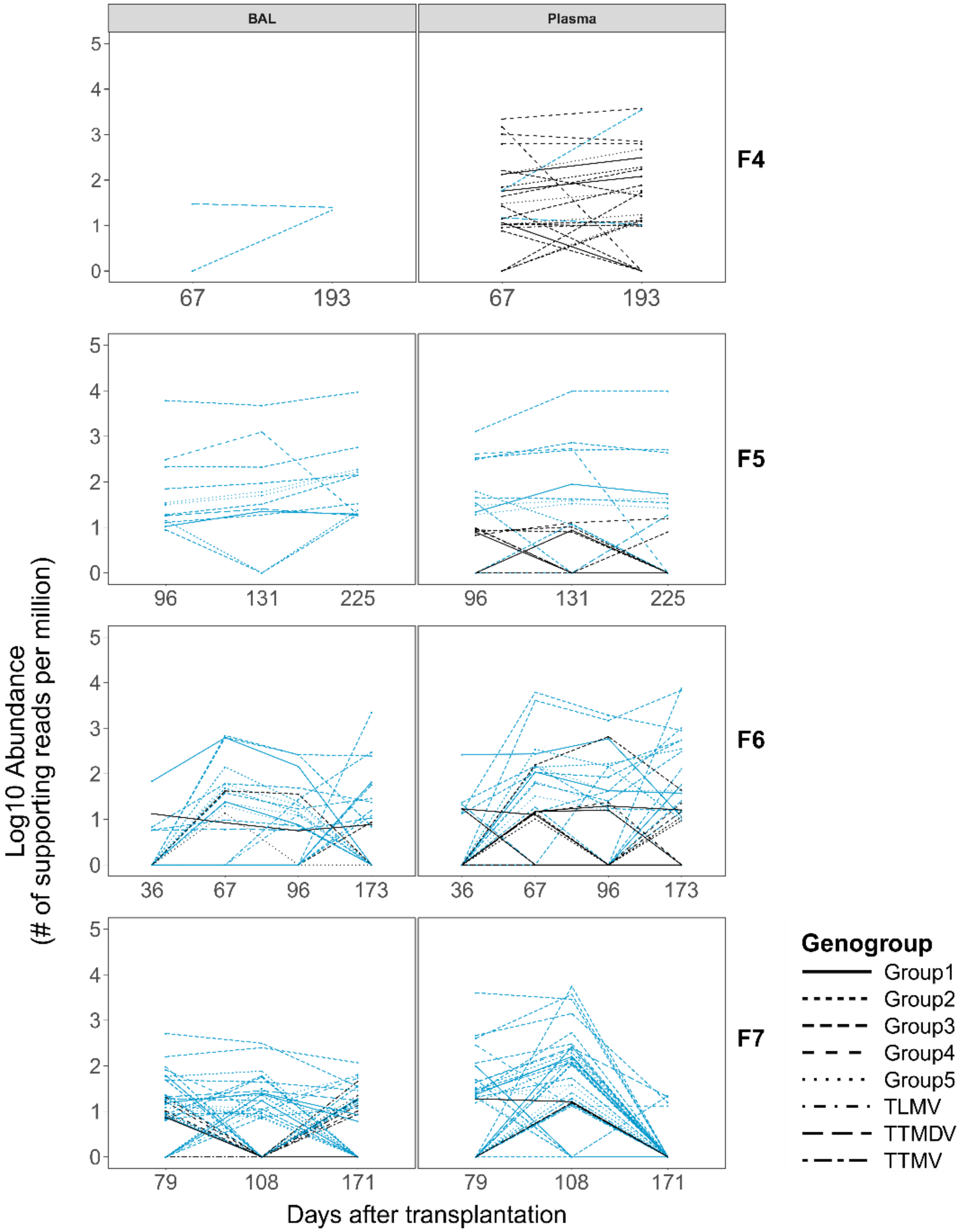

Figure S3. Anellovirus dynamics over time in 4 follow up lung transplant recipients (F4-F7). In blue the strains that occur both in BAL and plasma are displayed.
