# Supplementary material for: Temporal dynamics of the lung and plasma viromes in lung transplant recipients
Source: PLoS One. 2018 Jul 6;13(7):e0200428. doi: 10.1371/journal.pone.0200428 (PMC6034876; doi:10.1371/journal.pone.0200428)
Supplement: S5 Fig — The number of strains in each intersecting set is shown as vertical barplots and the total number of strains identified per time point is shown in blue horizontal barplots. (PDF) [file pone.0200428.s006.pdf]

Plasma samples

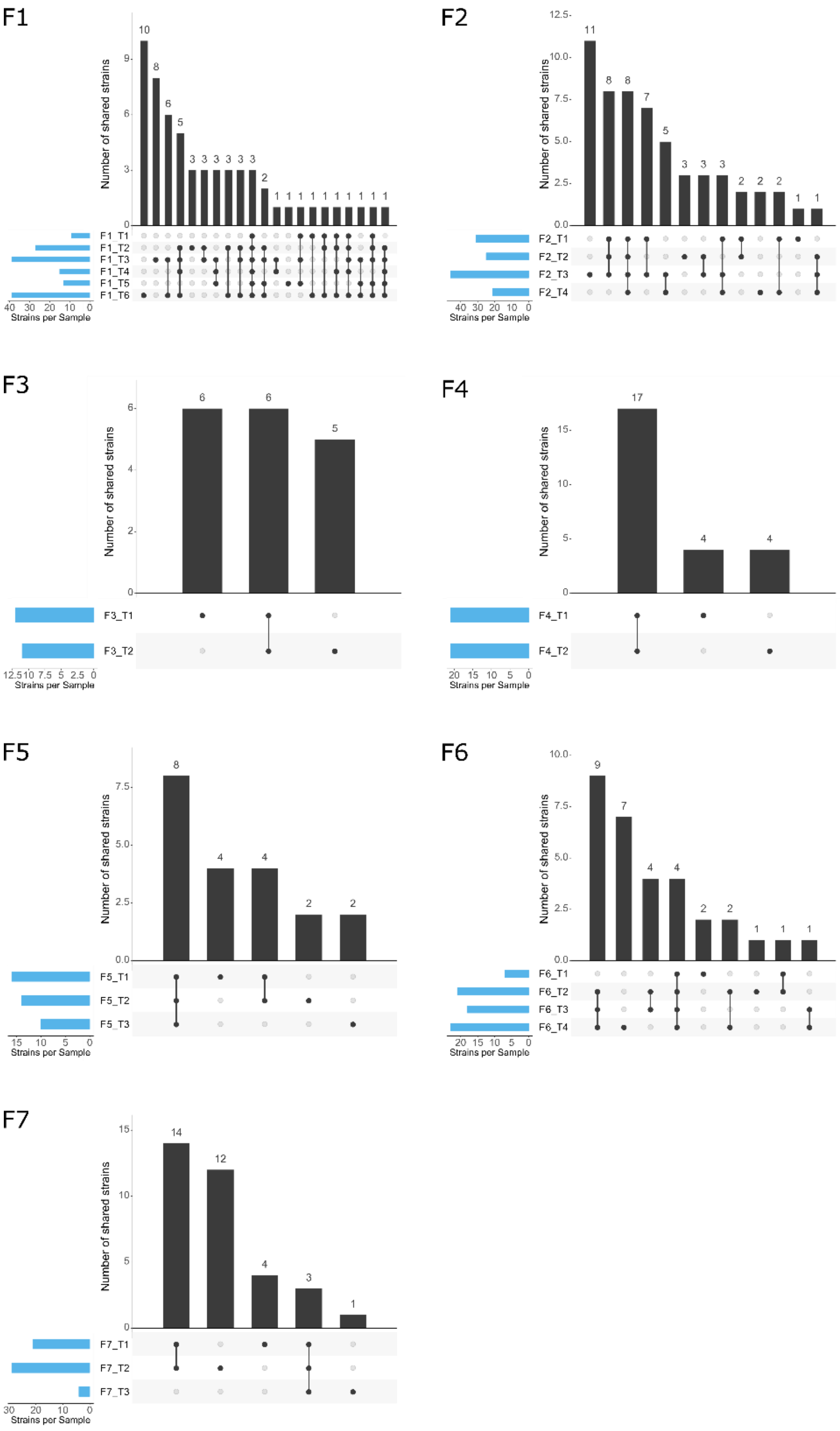

Figure S5. Number of anellovirus strains per patient and per time point identified in the plasma of LTRs. The number of strains in each intersecting set is shown as vertical barplots and the total number of strains identified per time point is shown in blue horizontal barplots.
