# Supplementary figures and images for: Temporal dynamics of the lung and plasma viromes in lung transplant recipients
Source: PLoS One. 2018 Jul 6;13(7):e0200428. doi: 10.1371/journal.pone.0200428 (PMC6034876; doi:10.1371/journal.pone.0200428)

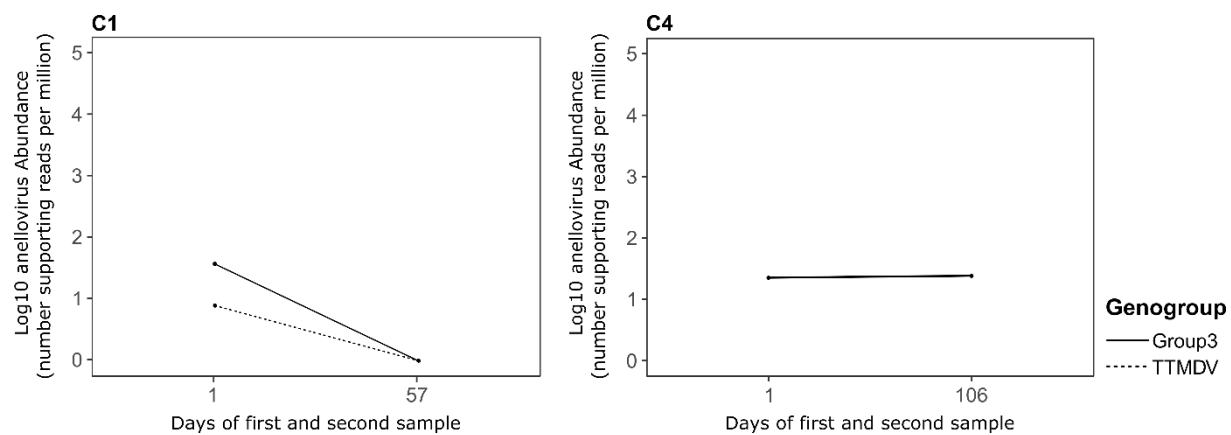

Figure S6. Anellovirus dynamics over time in two plasma samples obtained from healthy controls.

Supplement: S6 Fig — (PDF) [file pone.0200428.s007.pdf]
