# Supplementary material for: Temporal dynamics of the lung and plasma viromes in lung transplant recipients
Source: PLoS One. 2018 Jul 6;13(7):e0200428. doi: 10.1371/journal.pone.0200428 (PMC6034876; doi:10.1371/journal.pone.0200428)
Supplement: S7 Fig — Anellovirus diversity (measured by Shannon diversity index) in LTRs in relation to (A) underlying disease (CF: cystic fibrosis; COPD: chronic obstructive pulmonary disease), or (B) gender. (PDF) [file pone.0200428.s008.pdf]

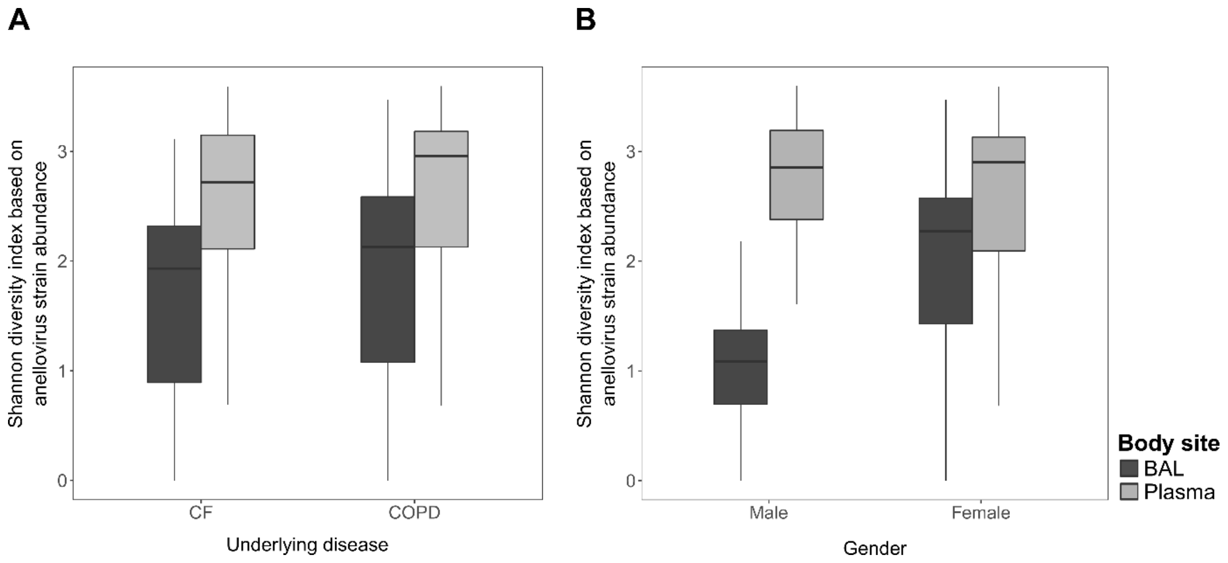

Figure S7. Anellovirus diversity (measured by Shannon diversity index) in LTRs in relation to (A) underlying disease (CF: cystic fibrosis; COPD: chronic obstructive pulmonary disease), or (B) gender.
